# Effects of Capsule Type on the Characteristics of Cement Mortars Containing Powder Compacted Capsules

**DOI:** 10.3390/ma15196773

**Published:** 2022-09-29

**Authors:** Se-Jin Choi, Sung-Ho Bae, Dong-Min Ji, Sung-Hoon Kim

**Affiliations:** 1 Department of Architectural Engineering, Wonkwang University, 460 Iksan-daero, Iksan 54538, Korea; 2 Department of Electronics Convergence Engineering, Wonkwang University, 460 Iksan-daero, Iksan 54538, Korea

**Keywords:** powder compacted capsule, cementitious materials, cement mortar, fluidity, compressive strength, carbonation depth, water permeability test

## Abstract

Several studies have been reported on self-healing concrete using bacteria, admixtures, and microcapsules. Among these self-healing techniques, encapsulating cement-based materials is advantageous in that large amounts of self-healing material can be contained in a capsule and released at the cracked site for a targeted reaction. This study produced a powder compacted capsule (PCC) using the droplet and blended manufacturing methods to encapsulate cementitious materials. This study refers to the PCCs as droplet-PCC (D-PCC) and blended-PCC (B-PCC) according to the manufacturing method used. The fluidity, compressive strength, carbonation, drying shrinkage, and water permeability of cement mortar with PCCs were evaluated. The test results show that the flow of the mortar sample using D-PCC was slightly higher than that of the mortar using B-PCC. The compressive strength of the mortar sample with B-PCC was generally higher than that of the mortar sample with D-PCC. The compressive strength of the B-PCC2 sample (with 0.2% of B-PCC) was the highest at all curing ages. This may be because the B-PCC fracture load was higher than that of the D-PCC. In addition, more hydrates were observed in the B-PCC sample than in the D-PCC sample. A crack healing effect was observed in the samples with PCC, regardless of the PCC type. The effect was the greatest in the B-PCC6 sample (with 0.6% of B-PCC). The results of this study provide a reference for the PCC type and mix ratio that would yield the best mechanical properties and crack healing effect.

## 1. Introduction

Concrete is a commonly used material in the construction industry owing to the easy production and processing of its raw materials, excellent formability, and excellent structural performance [1,2]. However, microcracks on the concrete surface caused by various external factors act as penetration paths for deterioration factors, promoting crack growth and lowering the concrete durability [3]. In addition, as deterioration progresses, the usability and durability of concrete facilities decrease, and repair costs increase. Crack repair may be difficult in underground structures [4].

Concrete can generally heal cracks by rehydrating the unhydrated cement particles and water in the concrete for microcracks. However, the healing effect is insignificant [5]. Several studies have been reported on self-healing concrete in which cracks are healed using bacteria, admixtures, and microcapsules [6,7,8,9,10,11,12,13,14]. Virginie et al. [6] reviewed the crack-healing effect of bacteria-based self-healing concrete. They reported that the crack-healing effect of bacteria-based self-healing concrete cured in water for 100 d doubled owing to the metabolic activity of the bacteria. Hu et al. [9] reviewed the performance of the optimized one-component polyurethane healing agent in self-healing concrete and reported that the recovery rate of flexural strength of concrete with glass capsules increased by approximately 6–30%. Wang et al. [11] reviewed self-healing concrete using microencapsulated bacterial spores. The healing rate of the samples with microcapsules was found to be higher than that of the samples without bacteria.

Among the discussed self-healing technologies, the encapsulation of cement-based materials is advantageous, specifically because large amounts of self-healing material can be contained in a capsule and released at the cracked site for a targeted reaction [15,16]. When the cementitious self-healing material is released from the crack area, it can react with the pore water in the cement composite to form hydrates, thereby producing a healing effect. In addition, if a capsule is not destroyed by cracks, the healing ability remains latent. Thus, the self-healing performance remains effective [17]. Several studies have been conducted on encapsulated-based self-healing technology in mortar and concrete [18,19,20]. Fang et al. [18] reviewed the efficiency of cementitious material capsules considering the effect of capsule thickness. The self-healing efficiency varied depending on the shape of the cylinder and spherical capsule.

Wu et al. [19] reviewed the self-healing performance of bacterial spores coated with inorganic cementitious materials. Crack sizes of 150 μm were healed using self-healing capsules with 5% of the amount of cement. Ren et al. [20] reviewed the performance of microcapsules according to the mixing temperature. The self-healing performance was observed to improve at a higher temperature as opposed to a lower temperature.

Typically, microfluidic channels develop droplets or capsules [21,22,23,24]. However, the microfluidic chip manufacturing process is time-consuming and requires expensive equipment. Moreover, in the case of a powder-mixed fluid, the microfluidic channel is easily clogged by the powder, hindering stable mass production. Therefore, to avoid these issues, a simple configuration droplet manufacturing method that provides high uniformity and precision without microchannels is proposed. Consequently, stable production of droplets mixed with a large amount of powder using the developed system is achieved. In particular, the proposed droplet manufacturing method is a new PCC manufacturing method that has not yet been applied.

This study produced a powder compacted capsule (PCC) using the droplet and blended manufacturing methods [15,25] to encapsulate a cementitious material. Moreover, the mechanical properties of the PCC were evaluated. Subsequently, the fluidity, compressive strength, carbonation, drying shrinkage, and water permeability of the cement mortar mixed with PCC were evaluated.

## 2. Materials and Methods

### 2.1. Materials

Ordinary Portland cement (Asia Co, Seoul, Korea) with a 28 day-compressive strength of 42.5 MPa was used in this study. Natural sand from Namwon (Korea) with a density of 2.60 g/cm^3^ and a fineness modulus of 2.45 was used as fine aggregate. The cementitious materials used in the PCC comprised ordinary Portland cement, fly ash (Dangjin Thermal Power Plant, Dangjin-si, Korea), and blast furnace slag powder (Daehan Slag, Gwangyang, Korea). Table 1 presents the chemical properties of the cementitious materials used in this study.

For droplet-PCC (D-PCC), the powder-mixed droplet solution was a mixture of UV resin (Clear) from Anycubic and cementitious powder (Cement, fly ash, and blast furnace slag powder in a 4:3:3 ratio). The continuous phase was a mixture of KF-96-1000cSt (50%) and KF-96-500cSt (50%) silicone oils. UV light (Prime-100 [365 nm 130 Deg]) from skycares was used to cure the droplets. The droplets produced were photographed using a digital microscope (Dino-Lite AM73115).

For the blended PCC (B-PCC), polyvinyl alcohol (PVA, Amos Co., Seoul, Korea) was stirred with the cementitious material to improve cohesion. The PCC surface was coated with polyurethane (Jevisco Co., Seoul, Korea) to inhibit premature hydration of the PCC.

### 2.2. Manufacturing Process of PCCs

#### 2.2.1. Droplet-PCC (D-PCC) 

**Figure 1:**
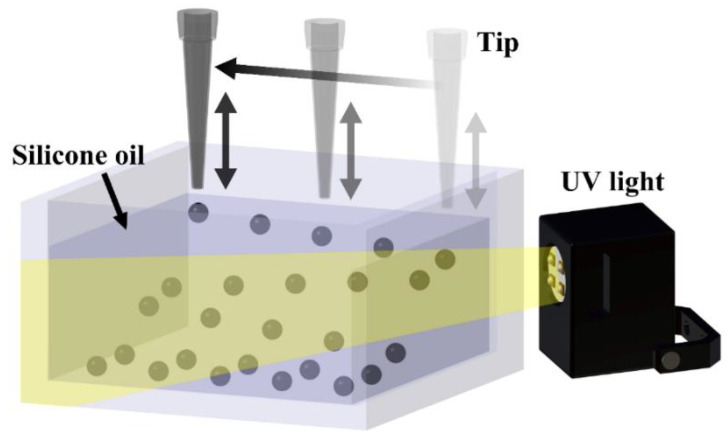
Schematic illustration for generation of the droplet.

The powder mixed droplet solution was prepared by mixing the powder into the resin at three different ratios of 10 wt%, 15 wt%, and 20 wt%, followed by stirring for 10 min. The droplet solution was extruded through a needle at a constant flow rate, and the droplet was separated on the surface of the silicone oil through the vertical movement of the needle. The droplet solution was extruded at a 1.1 mL/h flow rate, and vertical needle movement occurred every 6 s. The separated droplets moved to the bottom inside the silicone oil and were cured using UV light. UV light was irradiated from multiple directions by reflecting the source off of a mirror. For mass production, the needle from which the droplet solution was extruded was moved at regular intervals to prevent droplets from combining. Figure 1 shows a schematic of the droplet manufacturing system.

#### 2.2.2. Blended-PCC (B-PCC) 

The ratio of water to cementitious material was fixed to 15% through a prior experiment. A 1.2–2.5 mm B-PCC was prepared by mixing cement, fly ash, and blast furnace slag powder in a 4:3:3 ratio. The dry materials were mixed with a mechanical mixer, and the produced B-PCC was sorted into the required size by using a sieve. The prepared B-PCC was coated with polyurethane using a spray gun after 24 h.

**Figure 2:**
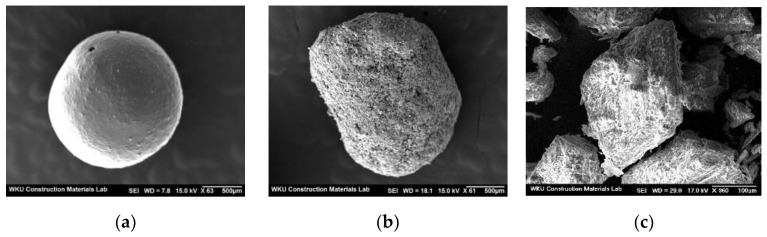
SEM images of PCC and fine aggregate: (**a**) D-PCC; (**b**) B-PCC; (**c**) fine aggregate.

Figure 2 shows the SEM images of the PCCs and fine aggregates.

### 2.3. Experimental Methods for Measuring Mechanical Properties of PCC

The size of the produced droplet-PCC was measured using a digital microscope. More than 70 droplets of each type were measured for uniformity, precision, and average size analysis. The average size, histogram size, and standard deviation were investigated using the MATLAB software.

**Figure 3:**
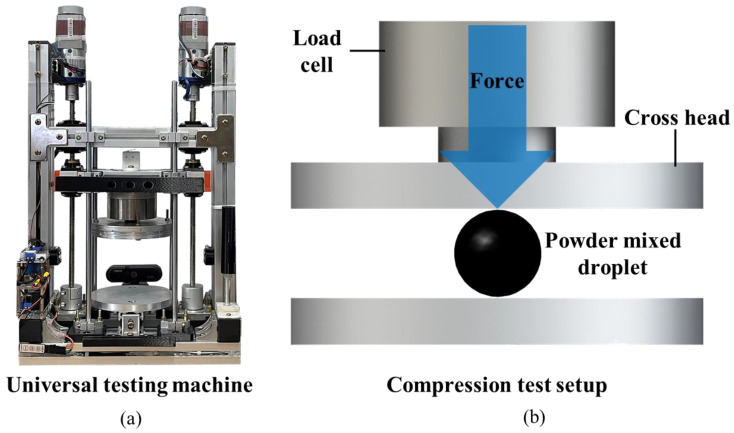
(**a**) A universal testing machine (UTM). (**b**) Schematic of droplet compression experiments.

The mechanical properties of the fabricated PCCs were investigated using a homemade lab-scale universal testing machine (UTM), shown in Figure 3a. The resolution of the load cell mounted on the UTM was 0.001 [N], and the measurement range was from 0 to 2000 N. The droplet was placed at the center of the lower plate, as shown in Figure 3b. The descending speed of the crosshead was set as 2 mm/min. The compressive strength test was performed four times for each PCC type.

### 2.4. Mixing Proportions and Specimen Preparation

Table 2 lists the mix proportions of the cement mortars. W/C was fixed at 50%, and each PCC was replaced by 0.2%, 0.4%, and 0.6% of the fine aggregate weight.

For the cement mortar mixed with PCC, 25 mm × 25 mm × 25 mm cubic specimens were prepared by molding for compressive strength testing, and 40 mm × 40 mm × 160 mm specimens were prepared for drying shrinkage testing. Additionally, 50 mm × 100 mm cylindrical specimens were prepared for accelerated carbonation testing, and 100 mm × 50 mm specimens were prepared for the water permeability test. Subsequently, the specimens were demolded after 24 h and cured in a water tank at 20 °C until they reached the required age.

The mortar flow and compressive strength were measured according to KS L 5105 [26] and the drying shrinkage was measured in a temperature-controlled room with a temperature of 20 ± 3 °C and a humidity of 60 ± 5% according to KS F 2424 [27]. The carbonation test measured the carbonation depth using a phenolphthalein solution after the carbonation process in an accelerated carbonation chamber according to KS F 2584 [28].

**Figure 4:**
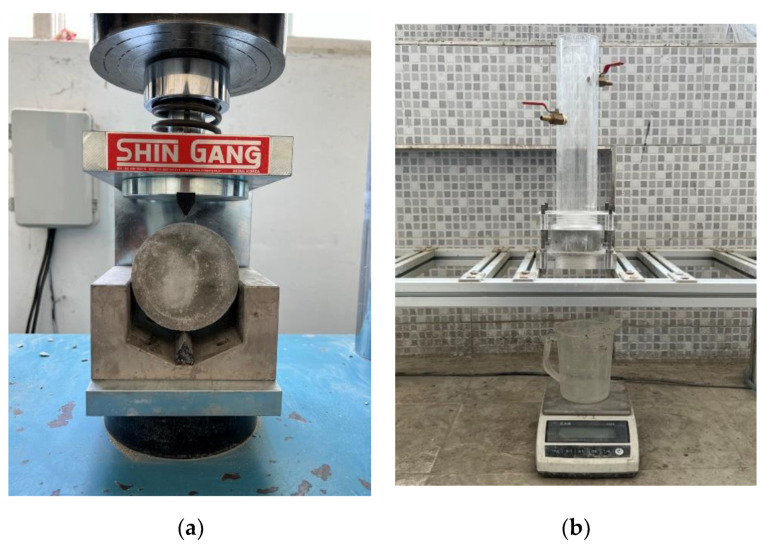
Water permeability test device: (**a**) specimen splitting; (**b**) measurement of the amount of flow water.

In the water permeability test, the specimen was split after 28 d of age, and 0.3 mm silicone sheets were placed on both ends of the specimen to maintain the crack width. After fixing the specimen using a clamp, a water permeability test (Figure 4) was performed at each age according to previous studies [11,15,29,30].

## 3. Results and Discussion

### 3.1. Mechanical Properties of PCCs

**Figure 5:**
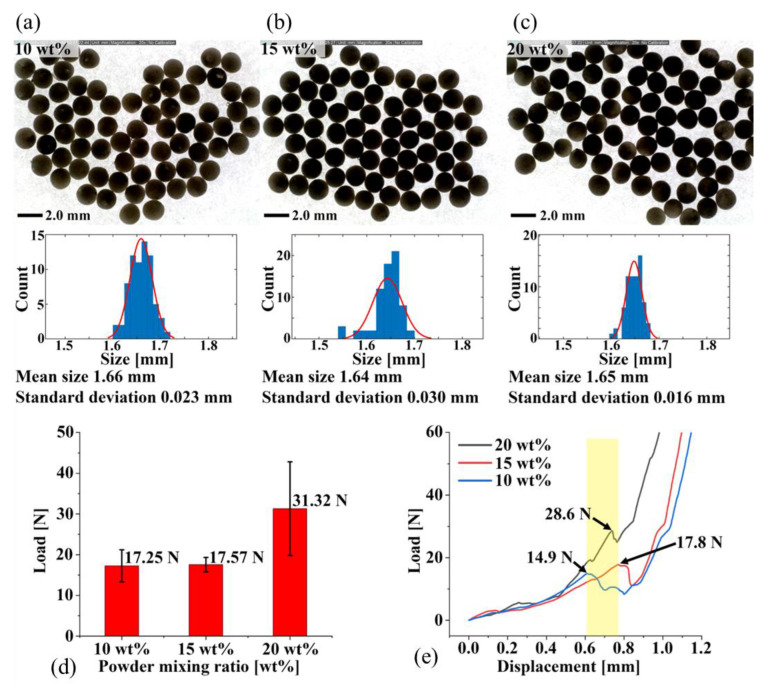
Drop size measurement and results of fracture test. Size analysis results at powder ratio (**a**) 10 wt%, (**b**) 15 wt% and (**c**) 20 wt%. (**d**,**e**) Compression test results at powder ratio 10 wt%, 15 wt%, and 20 wt%.

The size distribution of the three types of droplets was investigated to confirm the reproducibility of the droplet fabrication, as shown in Figure 5a–c. The average sizes of the droplets were 1.66 mm, 1.64 mm and 1.65 mm, respectively. The standard deviations of the droplet sizes were 0.023 mm, 0.03 mm and 0.016 mm, respectively, indicating a high precision. A droplet compression test was performed using a UTM to confirm the mechanical properties of the powder-mixed droplets. Compression tests were performed using three types of droplets (10 wt%, 15 wt% and 20 wt% of powder). The compressive strength increased with an increase in the powder ratio. The compressive strength at ratios of 10 wt% and 15 wt% was 17 N, and it increased significantly to 31 N at 20 wt%, as shown in Figure 5d. All three droplet types broke at displacements between 0.6 mm and 0.8 mm, as shown in Figure 5e. Table 3 lists the compression test results for the D-PCC samples. The fracture load values of the D-PCC samples were measured to be 13–21.2 N at 10 wt%, 15.9–19.9 N at 15 wt%, and 22.5–48.2 N at 20 wt%. Therefore, the powder ratio of D-PCC was determined to be 20 wt%.

Table 4 lists the compression test results for the B-PCC samples. The fracture load values of the B-PPP samples were measured to be about 13.85–78.33 N.

The average value was approximately 32.75 N, relatively higher than that of D-PCC. Moreover, the capsule collapsed when B-PCC was destroyed.

### 3.2. Mortar Flow

**Figure 6:**
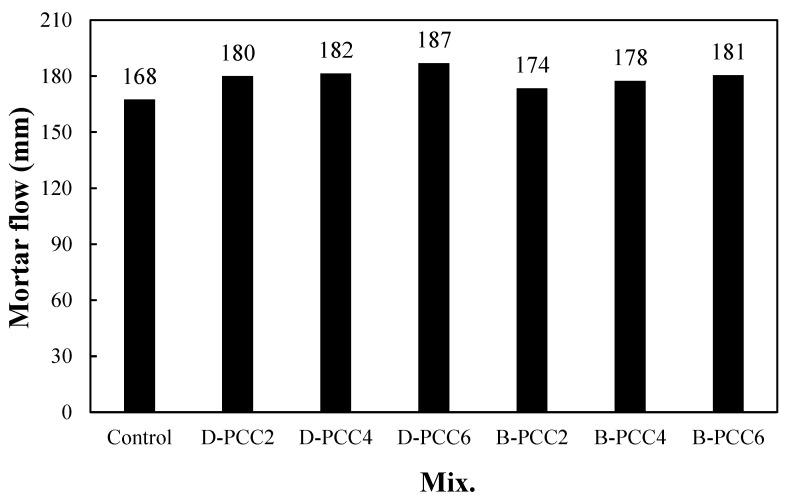
Mortar flow.

Figure 6 shows the flow change in the mortar using the PCC according to the capsule type. The flow of the control sample was the lowest at approximately 168 mm. The flow of the mortar sample using D-PCC was approximately 180–187 mm, approximately 7.1–11.3% higher than that of the control sample. Even in the mortar sample with B-PCC, the mortar flow was approximately 174 mm to 181 mm, approximately 3.5–7.7% higher than that of the control sample. This is because the two types of PCC particles (Figure 2) are spherical, unlike the fine aggregate.

In the mortar sample with the same amount of PCC, the flow of the mortar sample using D-PCC was slightly higher than that of the mortar sample using B-PCC. This is probably because the surfaces of the D-PCC particles are smoother than those of the B-PCC particles.

The mortar flow increased as the PCC addition rate increased for both types of PCC.

### 3.3. Compressive Strength

**Figure 7:**
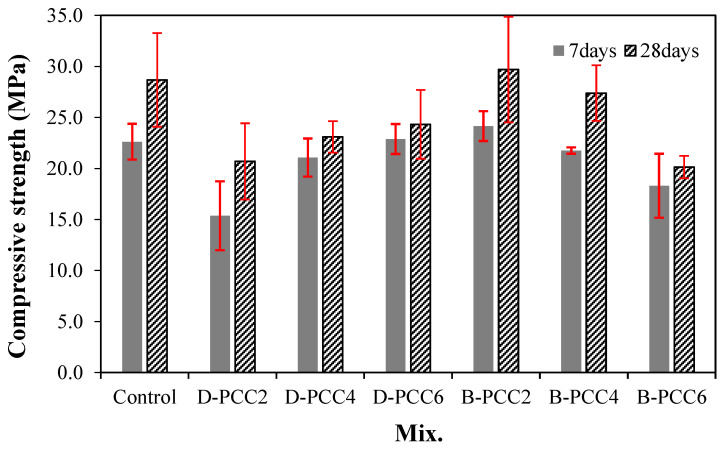
Compressive strength.

Figure 7 shows the change in the compressive strength of the mortar using PCC according to the capsule type. The 7-day compressive strength of the control sample was approximately 22.6 MPa.

In the case of the mortar sample using D-PCC, the compressive strength of the D-PCC2 sample was approximately 15.4 MPa, approximately 31.8% lower than that of the control sample. As the amount of PCC increased, the compressive strength of the mortar also increased. The 7-day compressive strength of the D-PCC6 sample was approximately 22.9 MPa, similar to that of the control sample.

In the case of the mortar sample containing B-PCC, the 7-day compressive strength of the B-PCC2 sample was approximately 24.1 MPa, approximately 6.6% higher than that of the control sample. The compressive strengths of the mortar samples decreased as the B-PCC addition rate increased.

The compressive strength development trend was dependent on the type and amount of PCC used. This may present benefits such as void filling and particle size adjustment of small PCC particles; however, it may also result in a decrease in adhesion strength owing to the relatively smooth PCC surface, when compared to natural fine aggregates, and the low breaking strength of the PCC particles.

After 28 d, the compressive strength of the control sample was approximately 28.7 MPa. The 28-day compressive strength of the mortar using D-PCC was approximately 20.7 MPa to 24.3 MPa, approximately 15.3–27.8% lower than that of the control sample. Similar to the 7-day compressive strength, the compressive strength of the mortar sample increased as the D-PCC addition rate increased. When B-PCC was used, the 28-day compressive strength of the B-PCC2 sample was approximately 29.7 MPa. This was the highest measured value, approximately 3.4% higher than that of the control sample. The 28-day compressive strength of the B-PCC6 sample was approximately 20.1 MPa, relatively lower than the other samples.

The compressive strength of the mortar sample with B-PCC was generally higher than that of the mortar sample with D-PCC. This might be due to the fracture load of the B-PCC being higher than that of the D-PCC. The compressive strength of the B-PCC2 sample with 0.2% of B-PCC was the highest at all curing ages.

However, the 28-day compressive strength of the sample using 0.6% PCC was approximately 15.3–29.9% lower than that of the control sample, regardless of the PCC type.

Therefore, using an appropriate PCC effectively improved the compressive strength of mortar.

In the future, the strength, shrinkage, and healing effects of cement mortar using larger amounts of PCC, or by substituting PCC into the binder, should be investigated.

### 3.4. Carbonation Depth

**Figure 8:**
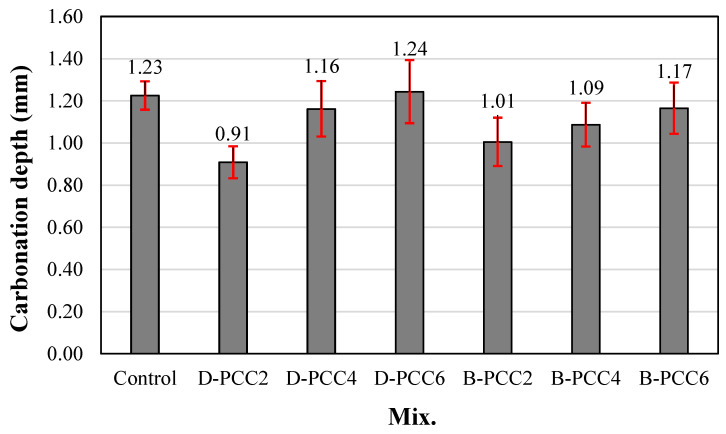
Carbonation depth.

Figure 8 shows the change in the carbonation depth of the mortar samples using PCC according to the capsule type after 28 d in the accelerated carbonation chamber. The carbonation depth of the control sample was 1.23 mm. The carbonation depth of the mortar sample using PCC increased as the amount of PCC used increased.

In the case of the sample using D-PCC, the carbonation depth of the D-PCC2 sample was approximately 0.91 mm, approximately 26% lower than that of the control sample. The carbonation depth of the sample using B-PCC was 1.09–1.17 mm, approximately 4.8–11.3% lower than that of the control sample. The carbonation resistance of the mortar sample containing PCC was better than that of the control sample. The carbonation resistances of the D-PCC2 and B-PCC2 samples using 0.2% PCC were higher than those of the samples using 0.4% and 0.6% PCC.

Owing to the PCC particles filling some of the pores in the cement composite, the carbonation resistance improved. The increase in the carbonation depth of the cement mortar samples, when the amount of PCC increased, could be a result of the infiltration of carbon dioxide at the interface between the PCC particles and cement paste. Significant carbonation has been shown to increase the number of voids surrounding the aggregate in cement composites, which may facilitate CO_2_ ingression [31,32].

### 3.5. Drying Shrinkage

**Figure 9:**
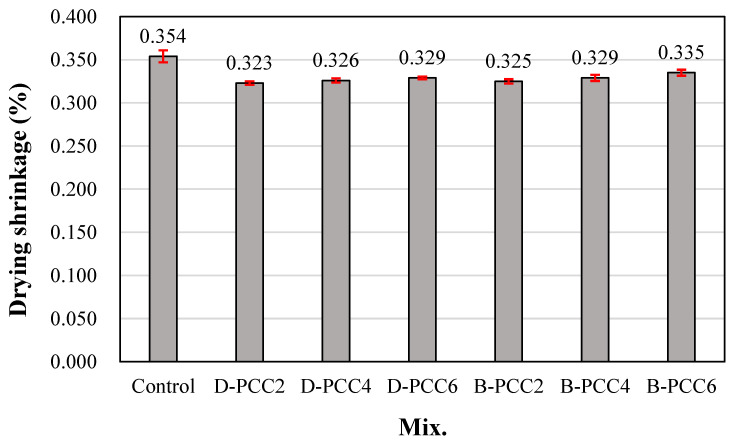
Drying shrinkage (56 days).

Figure 9 shows the change in the drying shrinkage of the mortar using PCC according to the capsule type. The drying shrinkage of the control sample after 56 d was the greatest at approximately 0.354%. The drying shrinkage of the sample using D-PCC was approximately 0.323–0.329%. The drying shrinkage of the sample using B-PCC was approximately 0.325–0.335%, which was similar regardless of the amount of PCC. The sample using D-PCC showed approximately 7.0–8.7% lower drying shrinkage than the control sample. The sample using B-PCC showed approximately 5.3–8.1% lower drying shrinkage than the control sample. The pore-filling effect of PCC is believed to have also affected the reduction in drying shrinkage of mortar. The difference according to PCC type and usage content was insignificant.

Therefore, it is necessary to investigate the strength, shrinkage, and healing effects of cement mortar using a larger amount of PCC, or by substituting PCC into the binder.

### 3.6. Water Permeability Test Result

**Figure 10:**
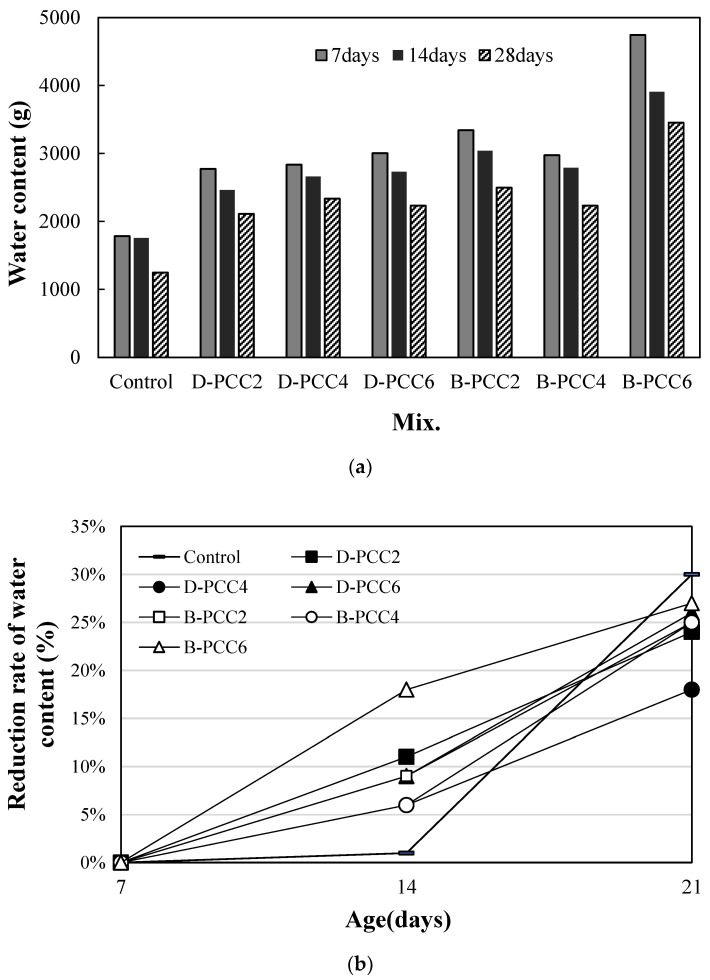
Water permeability test results: (**a**) water content passing through the crack; (**b**) reduction rate of the water content.

Figure 10 shows the water permeability test results for cracks in the mortar sample using PCC according to the capsule type. Measurements were performed at 7 d, 14 d, and 28 d after crack initiation.

The amount of water passing through the cracks in all samples decreased with age, as shown in Figure 10a.

Figure 10b shows the decrease rate of the amount of water passing through cracks based on 7 d. The decrease rate of the control sample at 14 d was the lowest at approximately 1%, as shown in Figure 10b. The reduction rates of all the samples using PCC were approximately 6–18%, larger than those of the control sample.

In the case of the sample using D-PCC, the decrease rate of the D-PCC2 sample was relatively large at approximately 11%. In the case of the sample using B-PCC, the reduction rate of the B-PCC6 sample was approximately 18%, the largest compared with the other samples.

Therefore, a crack healing effect was observed in the samples with PCC, regardless of the PCC type. Moreover, the effect was the greatest in the B-PCC6 sample. This trend continued even after 28 d. The decrease rate of the B-PCC6 sample was approximately 27%, the largest among the samples using PCC. Except for the D-PCC4 sample (18%), the reduction rates of all samples using PCC were approximately 24–27%, showing a relatively high value.

In addition, after 28 d, the reduction rate of the control sample was approximately 30%, showing a significant value. This may be because the initial amount of water passing through the crack of the control sample was the lowest (Figure 10a), rather than the crack-healing effect.

Further studies on the healing effect and microstructure analysis according to the PCC size, crack size, and water permeability are required in the future.

## 4. Conclusions

(1) The powder droplet manufacturing system can easily change droplet size by controlling the flow rate. Therefore, it provides high precision and uniformity for droplet fabrication. Furthermore, mass production can be performed using this simple manufacturing method. Moreover, this method has considerable economic advantages.
(2) Droplets containing 10 wt% and 15 wt% of powder showed similar properties in the compressive strength test. By contrast, droplets containing 20 wt% powder showed a high compressive strength of at least 14 N. The average load of the B-PCC was approximately 32.75 N, relatively higher than that of the D-PCC.
(3) In the mortar samples using the same amount of PCC, the flow of the mortar sample using D-PCC was slightly higher than that of the mortar using B-PCC. This is probably because the surfaces of the D-PCC particles are smoother than those of the B-PCC particles.
(4) The compressive strength of the mortar sample with B-PCC was generally higher than that of the mortar sample with D-PCC. The compressive strength of the B-PCC2 sample with 0.2% of B-PCC was the highest at all curing ages. Therefore, the use of an appropriate PCC was effective in improving the compressive strength of the mortar.
(5) The carbonation resistance of the mortar sample containing PCC was better than that of the control sample. The carbonation resistances of the D-PCC2 and B-PCC2 samples using 0.2% PCC were higher than those of the samples using 0.4% and 0.6% PCC.
(6) The amount of water passing through the cracks in all samples decreased with age. The reduction rate of the water content of all the samples using PCC was approximately 6–18%, larger than that of the control sample. Therefore, a crack healing effect was observed in the samples with PCC, regardless of the PCC type. The effect was the greatest in the B-PCC6 sample.

Further studies on the healing effect and microstructure analysis considering factors such as PCC size, crack size, and water permeability are required to comprehensively understand the applicability of PCC for improving the safety and serviceability standards of concrete structures.

## Figures and Tables

**Table 1:** Chemical composition of cementitious materials.

Type	SiO_2_	Al_2_O_3_	Fe_2_O_3_	CaO	MgO	K_2_O	Blaine (cm^2^/g)	Density (g/cm^3^)
Cement	17.43	6.50	3.57	64.40	2.55	1.17	3430	3.15
Blast furnace slag powder	30.61	13.98	0.32	40.71	6.43	0.60	4210	2.93
Fly ash	64.88	20.56	6.06	2.58	0.80	1.45	3710	2.21

**Table 2:** Mix proportion.

Mix	D-PCC (S*%)	B-PCC (S*%)	W/C (%)	Water (kg/m^3^)	Cement (kg/m^3^)	Sand (kg/m^3^)	PCC (kg/m^3^)
Control	-	-	50	170	340	739.0	-
D-PCC2	0.2	-			340	737.5	1.5
D-PCC4	0.4	-			340	736.0	3.0
D-PCC6	0.6	-			340	734.5	4.5
B-PCC2	-	0.2			340	737.5	1.5
B-PCC4	-	0.4			340	736.0	3.0
B-PCC6	-	0.6			340	734.5	4.5

**Table 3:** Compression test results of D-PCC.

Powder Ratio (wt%)	Test 1 (N)	Test 2 (N)	Test 3 (N)	Test 4 (N)
10	14.9	13	19.9	21.2
15	17.8	19.9	16.7	15.9
20	28.6	48.2	26	22.5

**Table 4:** Compression test results of B-PCC.

Sample No.	Displacement (mm)	Load (N)
Sample 1	0.297	28.33
Sample 2	0.519	34.95
Sample 3	0.289	31.53
Sample 4	0.333	45.67
Sample 5	0.154	16.69
Sample 6	0.128	22.91
Sample 7	0.220	34.55
Sample 8	0.122	13.85
Sample 9	0.298	78.33
Sample 10	0.218	20.75
Average	0.258	32.75

## Data Availability

Not applicable.
